# Coexistence of Suspected Tetralogy of Fallot With Absent Pulmonary Valve and Dextrocardia in Pre‐Gestational Diabetes Mellitus: A Case Report

**DOI:** 10.1002/ccr3.73597

**Published:** 2026-09-25

**Authors:** Alireza Golbabaei, Vida Kahani, Marzieh Mousivand

**Affiliations:** ^1^ Shariati Hospital Tehran University of Medical Sciences Tehran Iran; ^2^ Students Research Committee Khomein University of Medical Sciences Khomein Iran

**Keywords:** case report, pre‐gestational diabetes mellitus, prenatal diagnosis, Tetralogy of Fallot

## Abstract

Early fetal echocardiography and serial surveillance are crucial in pregnancies complicated by pregestational diabetes. Tetralogy of Fallot with absent pulmonary valve syndrome may cause progressive cardiomegaly, ventricular hypertrophy, hydrops, and fetal demise. Timely diagnosis enables informed parental counseling and multidisciplinary perinatal planning.

## 1 Introduction

Absent pulmonary valve syndrome (APVS) is a rare and serious congenital cardiac anomaly characterized by absence or severe dysplasia of the pulmonary valve, leading to marked pulmonary artery dilation and frequently accompanied by a large ventricular septal defect and right‐heart volume overload [1]. Although precise incidence rates are difficult to define, recent studies provide useful approximations that underscore the rarity of the condition [2].

In a comprehensive review of prenatally diagnosed cases, one series reported that APVS accounted for approximately 0.2%–0.4% of all congenital heart disease (CHD) cases [3, 4]. Among patients with Tetralogy of Fallot (TOF), the form most commonly associated with APVS, the incidence of APVS is often cited as 3%–6%, with some reports quoting 3% of TOF patients having the absent pulmonary valve variant [5]. In a recent tertiary‐centre series the incidence was specified as about 2% of TOF patients [6].

Beyond incidence, prenatal outcome data highlight the significant morbidity and mortality associated with APVS. For fetuses diagnosed before birth, a meta‐analysis found that of 199 cases across seven studies, 84.4% were associated with TOF [3, 7]. Another series of 37 prenatally diagnosed fetuses reported a survival after initial diagnosis of only 36.6% (11/30) overall; specifically 30% for TOF‐APVS and 40% for APVS with an intact ventricular septum (IVS) [8]. These data emphasize that while APVS remains rare, when it is present especially in the fetal period the prognosis is guarded.

The aim of this study is to present a rare case of fetal absent pulmonary valve syndrome associated with dextrocardia and maternal pre‐gestational diabetes, highlighting its prenatal echocardiographic findings, progression, and outcome. This report seeks to contribute to the growing understanding of the prenatal natural history and prognostic implications of APVS.

## 2 Case Presentation

A 33‐year‐old woman, gravida 2, para 0, abortus 1, with one living child, was referred to our clinic at 24 weeks of gestation for fetal cardiac evaluation. The patient had a history of pre‐gestational diabetes mellitus with suboptimal glycemic control (HbA1c 8.3% at the time of admission); no additional maternal risk factors were identified.

## 3 Methods and Treatment

**FIGURE 1:**
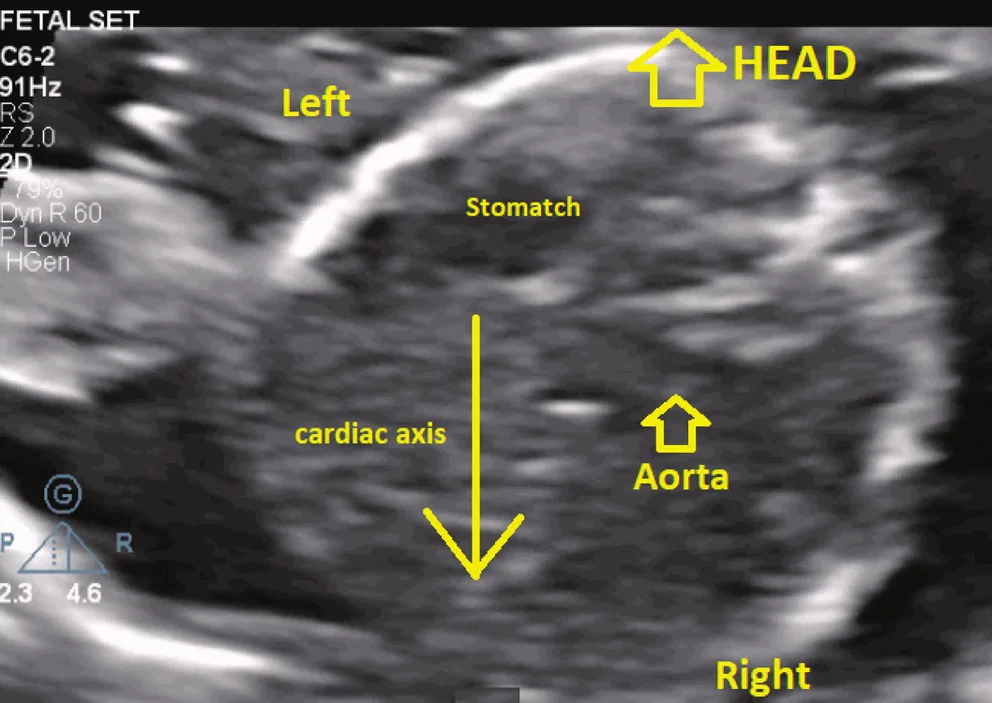
Axial fetal echocardiographic image demonstrates situs solitus with dextrocardia, showing rightward cardiac apex orientation.

**FIGURE 2:**
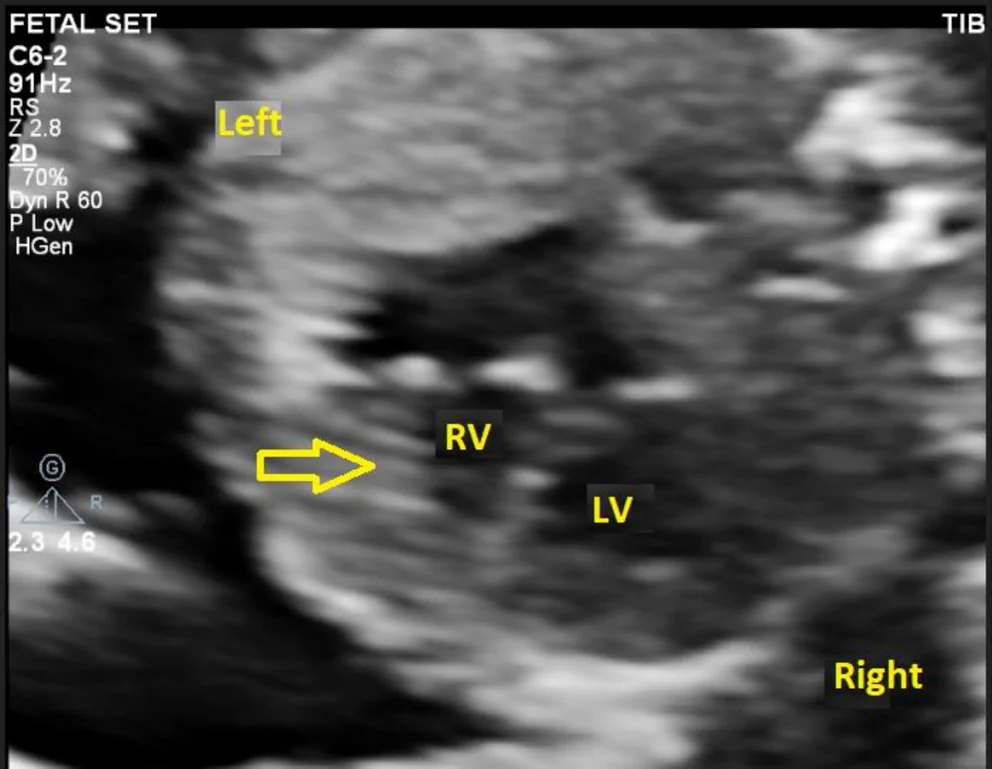
Axial echocardiographic view demonstrates dextrocardia with ventricular inversion and right ventricular free wall hypertrophy (yellow arrow).

**FIGURE 3:**
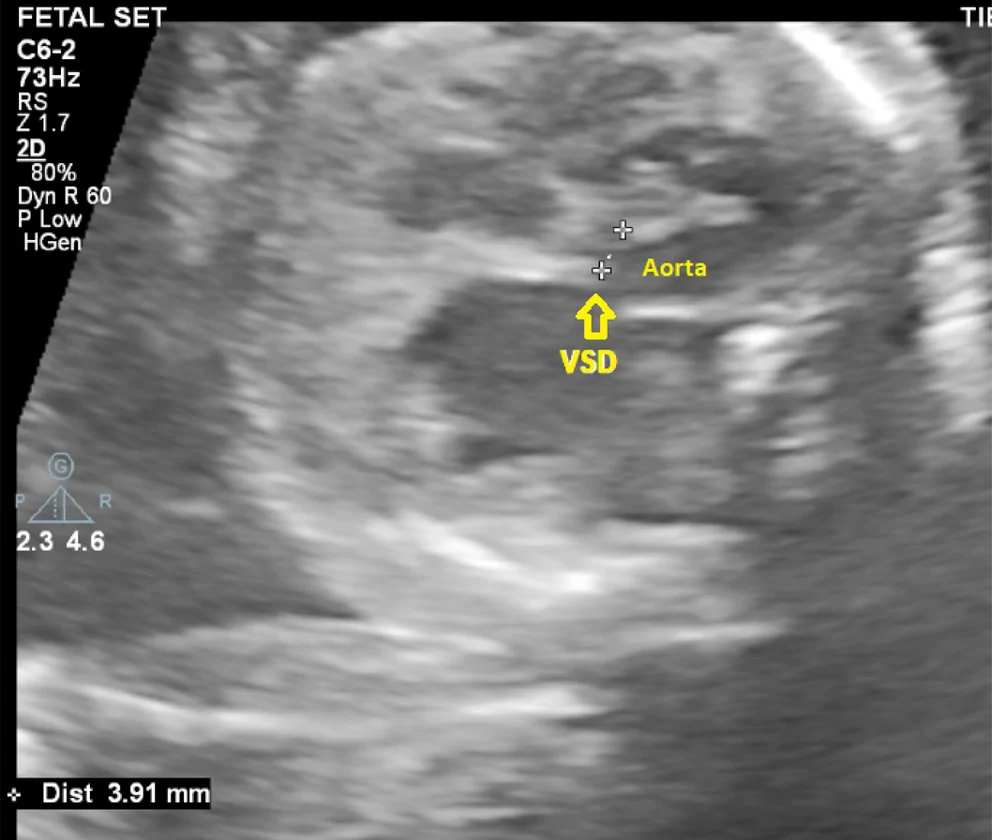
Left ventricular outflow tract view demonstrates a large subaortic ventricular septal defect (VSD) with overriding aorta, consistent with a conotruncal malformation.

**FIGURE 4:**
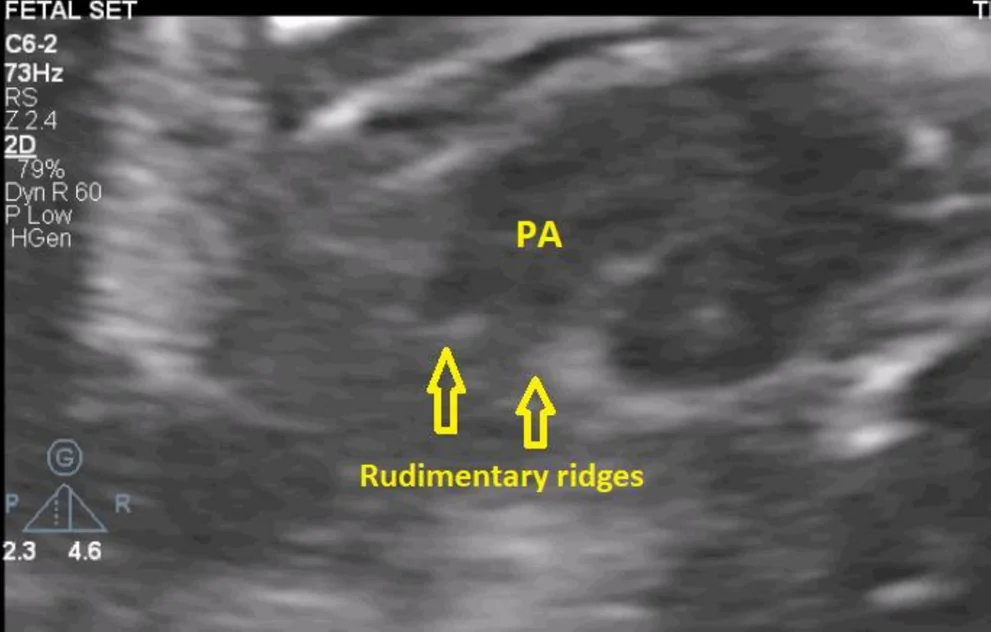
Right ventricular outflow tract view demonstrates rudimentary ridges with absence of normal pulmonary valve leaflets, suggestive of the absent pulmonary valve variant.

**FIGURE 5:**
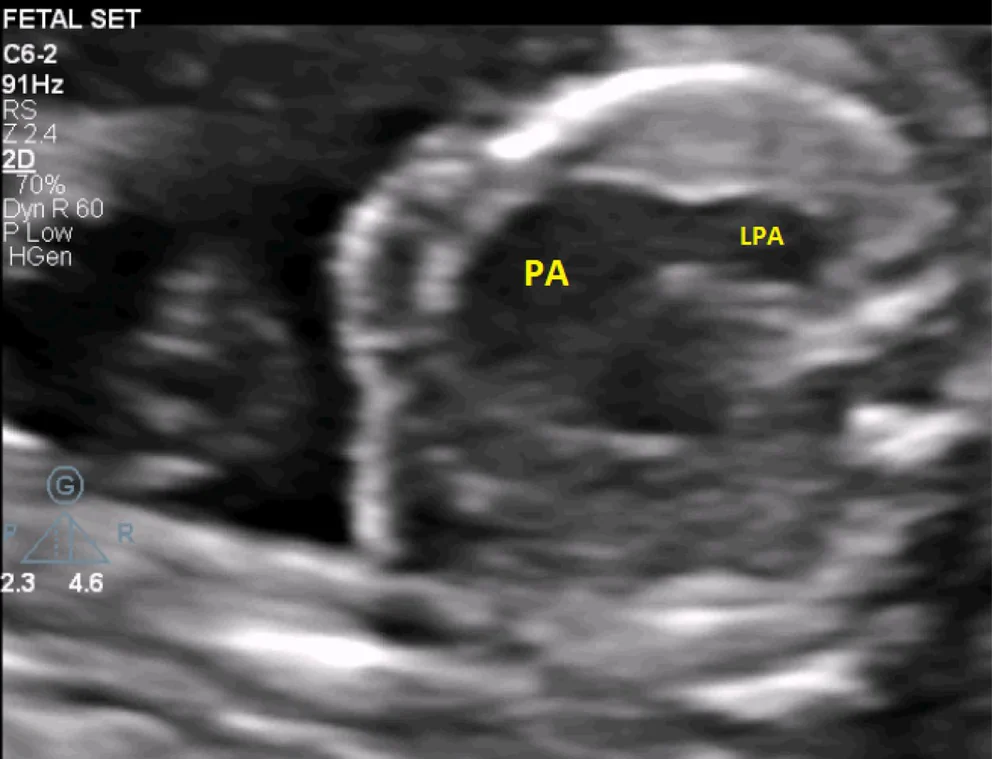
Axial view of the main pulmonary artery (PA) demonstrates marked dilation of the vessel and its left branch (LPA), with non‐visualization of the right pulmonary artery (RPA).

**FIGURE 6:**
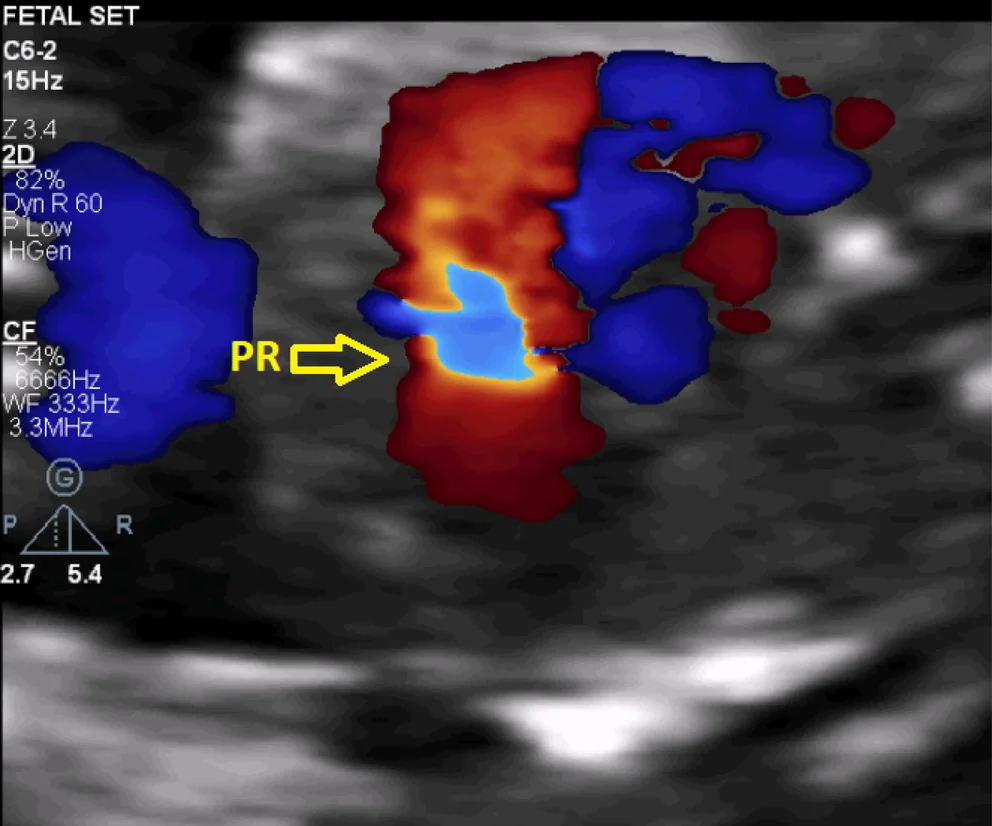
Color Doppler imaging in the axial view demonstrates significant pulmonary regurgitation (PR) with diastolic retrograde flow in the main PA.

**FIGURE 7:**
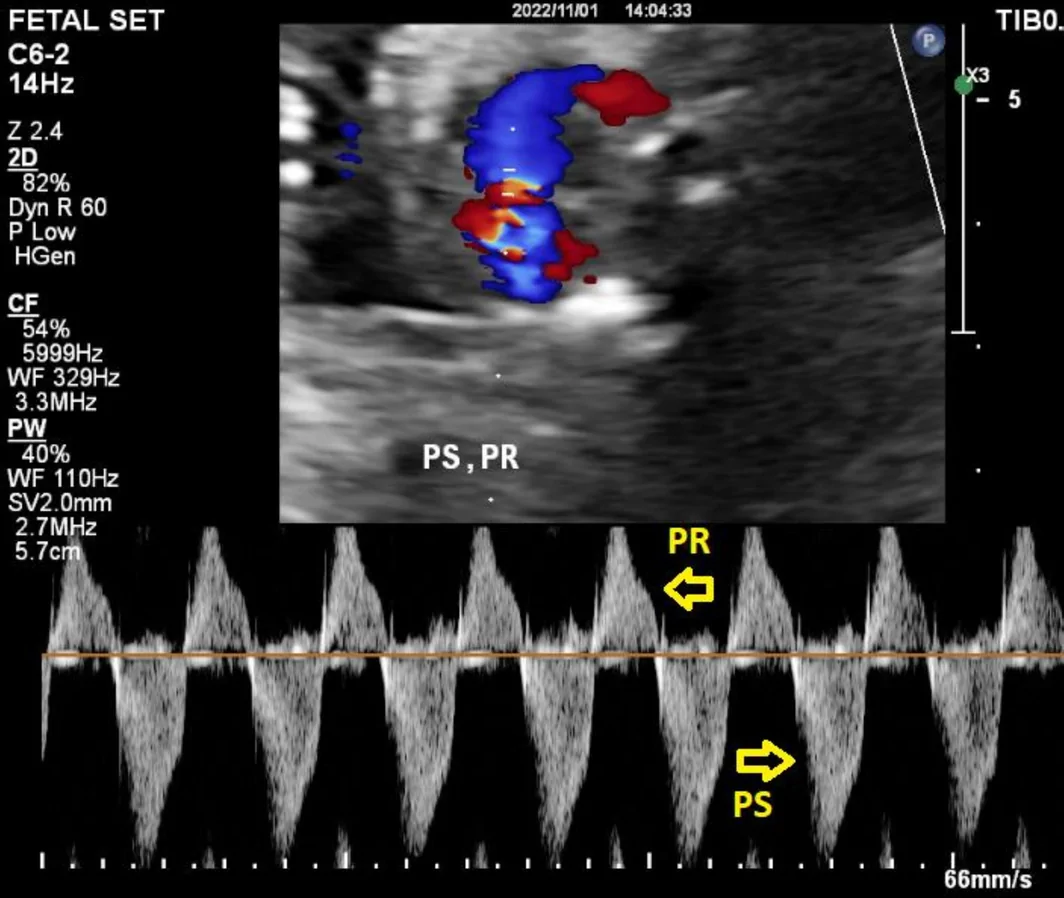
Spectral Doppler tracing of the pulmonary artery demonstrates severe pulmonary stenosis (PS) with prominent regurgitant diastolic flow (PR).

**FIGURE 8:**
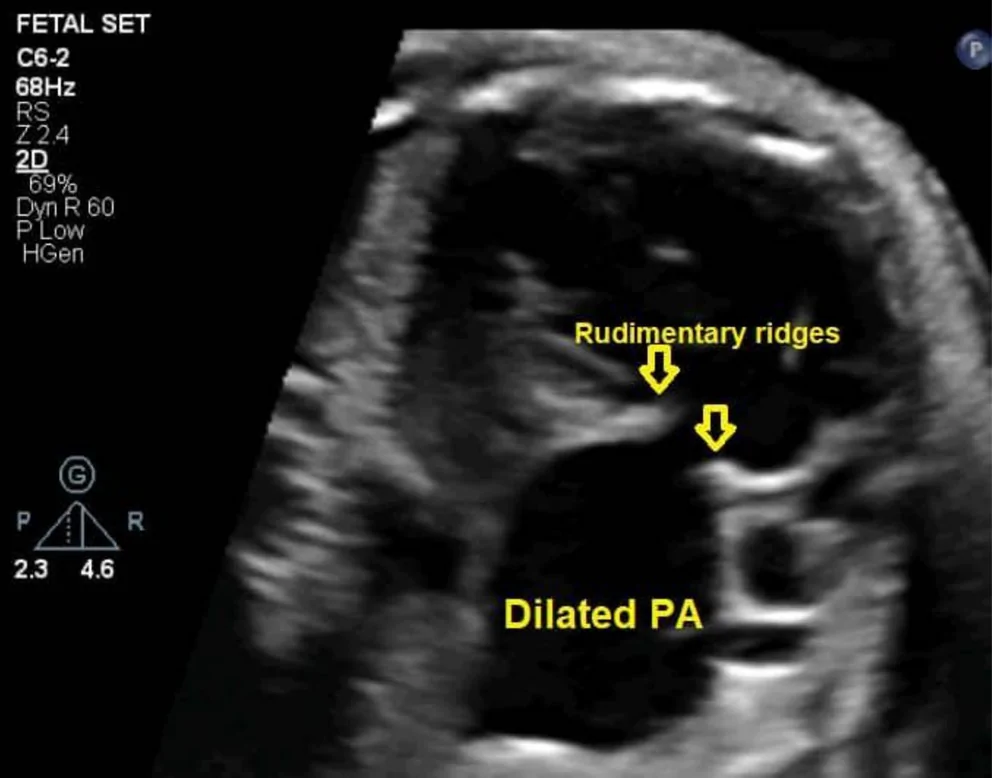
Dilated pulmonary artery with rudimentary pulmonary valve ridges (arrows).

A detailed fetal echocardiographic assessment was performed using standard two‐dimensional (2D), M‐mode, color Doppler, and spectral Doppler modalities. Initial evaluation demonstrated situs solitus with dextrocardia, consistent with isolated dextrocardia (Figure 1). Comprehensive Doppler interrogation also showed marked dilatation of the main pulmonary artery as well as pulmonary stenosis and pulmonary regurgitation (Table 1). In the apical four‐chamber view, the right ventricular free wall appeared hypertrophied (Figure 2), and subsequent left ventricular outflow tract imaging revealed a large sub‐aortic ventricular septal defect (VSD) accompanied by aortic overriding, findings compatible with a conotruncal anomaly (Figure 3). At this stage, there were no signs of hydrops fetalis, and umbilical Doppler indices remained within normal limits (Figures 4, 5, 6, 7). The Morphology of the pulmonary valve is shown in the Figure 8.

**TABLE 1:** Data regarding pulmonary artery dimensions.

Gestational age (GA)	Pulmonary artery dimension
24 weeks	15 mm
26 weeks	18 mm
28 weeks	21 mm

*Note:* CTR calculation method: Calculation of the ratio of the linear distance drawn between outer myocardial walls of the ventricles at the level of atriopulmonary valves to distance between the right and left sides of the thorax, at a 45 degree angle. The thickness of ventricular walls was assessed visually (no quantitative method was used for its measurement).

**FIGURE 9:**
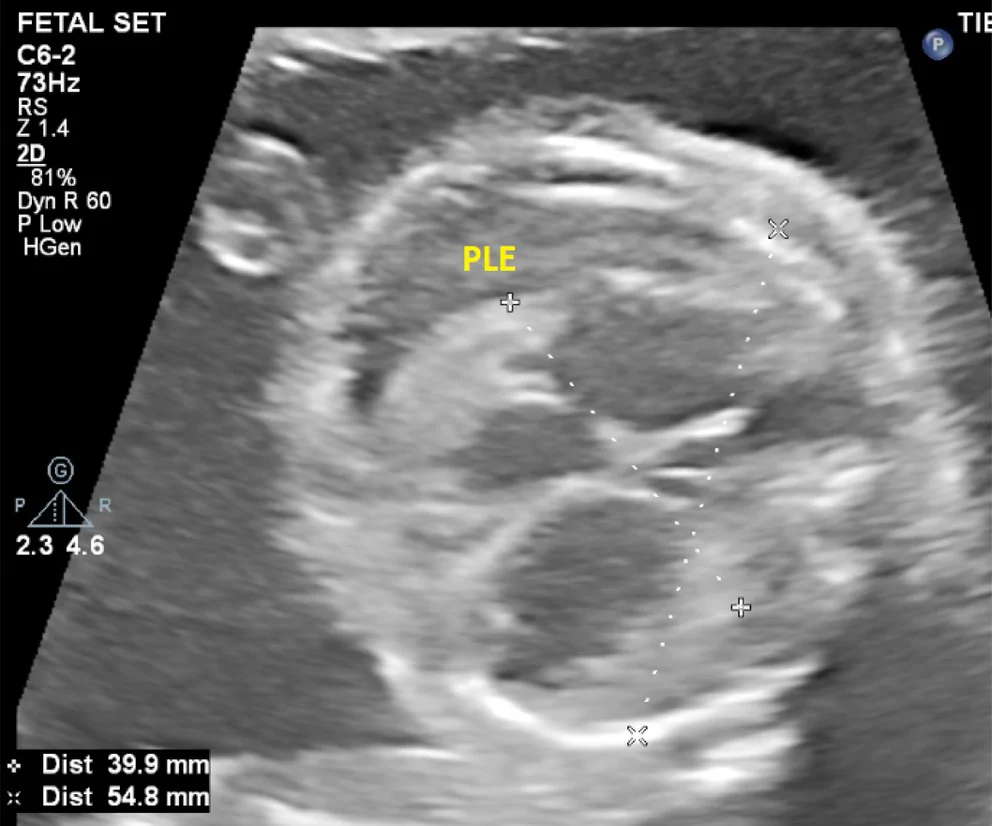
Follow‐up axial echocardiographic view obtained several weeks later demonstrates massive cardiomegaly, biventricular hypertrophy, and bilateral pleural effusions (PLE).

**FIGURE 10:**
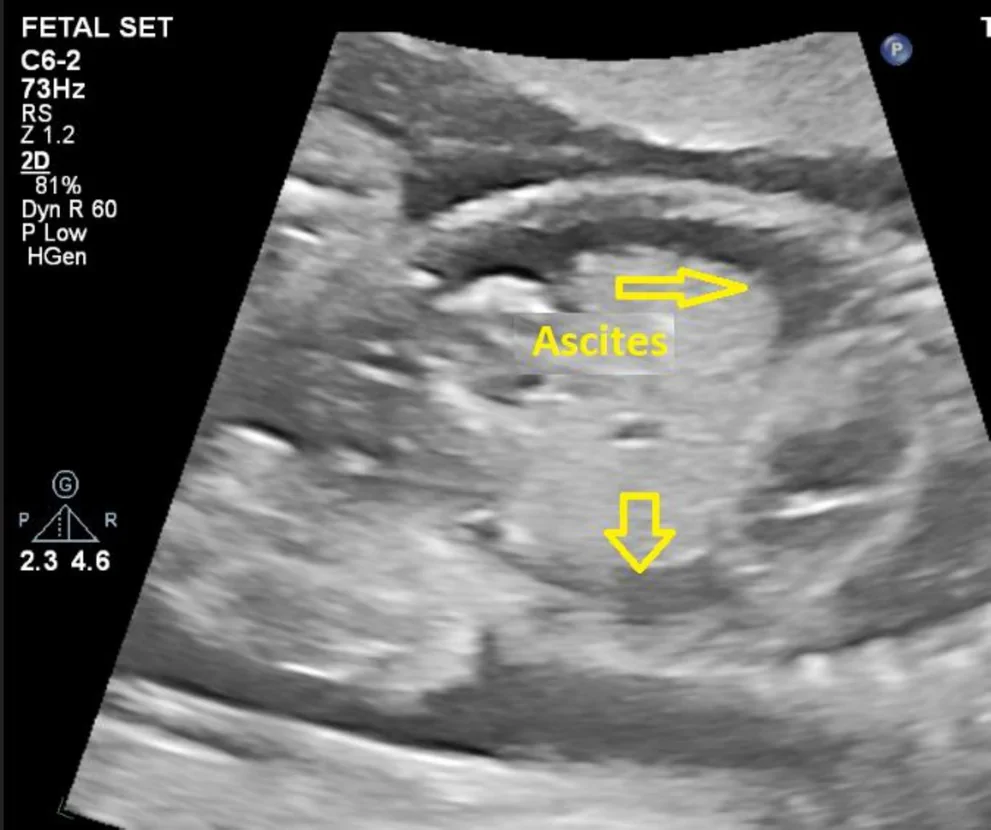
Sagittal sonographic view of the fetus demonstrates marked ascites (yellow arrows), consistent with evolving hydrops fetalis.

Serial fetal echocardiography was performed to monitor the progression of structural and hemodynamic abnormalities. On a follow‐up scan several weeks later, the fetus demonstrated significant progression of cardiac disease, characterized by massive cardiomegaly, biventricular hypertrophy, and the new onset of hydrops fetalis, including pleural effusion and ascites (Figures 9 and 10). Hydrops fetalis developed at 27 week of gestation. These findings indicated worsening cardiac compromise and poor fetal prognosis. A Serial fetal echocardiography demonstrating progressive cardiomegaly, ventricular hypertrophy, and Doppler abnormalities is shown in the Table 2.

**TABLE 2:** Serial fetal echocardiography demonstrating progressive cardiomegaly, ventricular hypertrophy, and Doppler abnormalities.

Gestational age (GA)	Cardiothorasic ratio (CTR)	Ventricular hypertrophy	Hydrops fetalis	Doppler velocity
24 weeks	70%–75%	Significant RVH	Was not observed	PS (80 cm/s)/PR (85 cm/s)
26 weeks	75%	Biventricular hypertrophy	Was not observed	PS (82 cm/s)/PR (91 cm/s)
28 weeks	75%–80%	Biventricular hypertrophy	Was seen	PS (81 cm/s)/PR (94 cm/s)

## 4 Conclusion and Result

Given the severity and progressive nature of the congenital heart defect, management consisted of close surveillance, including repeat echocardiography and counseling of the parents regarding prognosis and potential perinatal outcomes. No intrauterine cardiac intervention was deemed feasible due to the complexity of the defects and rapidly evolving hydrops. The pregnancy progressed until 30 weeks of gestation, at which time intrauterine fetal demise (IUFD) occurred secondary to the progression of hydrops fetalis despite maximal expectant management. At this time, the patient's HbA1C was 6.6%. Importantly, amniocentesis was not technically feasible because of the patient's delayed presentation to the hospital. Although noninvasive prenatal testing (NIPT) showed no evidence of trisomy 13, 18 or trisomy 21. Finally, progressive cardiomegaly, biventricular hypertrophy, and hydrops fetalis developed, culminating in intrauterine fetal demise at 30 weeks. Written informed consent from the patient was obtained for publication according to journal guidelines.

## 5 Discussion

The presented case had isolated dextrocardia, a large sub‐aortic VSD with aortic overriding, and RVOT obstruction with pulmonary regurgitation features consistent with a conotruncal lesion on the tetralogy‐of‐Fallot (TOF) spectrum. Isolated dextrocardia is very frequently accompanied by complex intracardiac defects (reported in ~90% + of cases), which carries a worse prognosis than mirror‐image dextrocardia [9].

The mother's pre‐gestational diabetes is a well‐established non‐inherited risk factor for CHD, with particularly strong associations for conotruncal defects. Hyperglycemia during cardiac morphogenesis promotes oxidative stress that impairs cardiac neural crest cell survival/migration and endocardial cushion development mechanisms repeatedly demonstrated in experimental models and human observational work [10, 11].

The markedly dilated pulmonary artery with pulmonary regurgitation raises concern for the absent pulmonary valve (APV) variant of TOF, a phenotype described with dextrocardia and known to cause severe cardiomegaly and respiratory compromise from massively dilated PA branches. Although we lacked postmortem confirmation, the Doppler pattern and progressive cardiomegaly/hydrops are concordant with that physiology [12].

Ultimately the fetus developed hydrops fetalis (pleural effusions, ascites) and intrauterine demise at 30 weeks of gestation. Cardiac hydrops reflects decompensated high central venous pressures and/or ventricular dysfunction and is a powerful adverse prognostic marker in fetal CHD [13].

This case underscores why targeted early fetal echocardiography (typically 18–22 weeks, with earlier assessment if feasible) is recommended for pregnancies at elevated CHD risk, including those with pre‐gestational diabetes. Early diagnosis enables: (i) comprehensive anatomic and rhythm assessment; (ii) serial surveillance for evolving ventricular hypertrophy, outflow obstruction, or signs of heart failure/hydrops; (iii) genetic counseling/testing (e.g., 22q11 deletion); and (iv) multidisciplinary delivery planning at tertiary centers. In cohorts of fetal dextrocardia and complex CHD, early prenatal diagnosis was pivotal for counseling and outcome stratification [14].

Once hydrops appears, outcomes deteriorate sharply in structural heart disease, and there are limited in utero therapeutic options for TOF/APV physiology beyond close hemodynamic monitoring and optimizing maternal–placental conditions. Reported series show high fetal/neonatal loss once hydrops supervenes, aligning with this course [13].

The most effective intervention to prevent cases like this is before conception. For example, tight glycemic control and maternal education before pregnancy lowers the risk and severity of fetal anomalies [15]. On the other hand, meta‐analyses and cohort studies show CHD risk rises stepwise with higher maternal HbA1c; compared with normal HbA1c (≤ 6%–6.5%), values > 8% confer ~4‐fold higher risk of major CHD, and even modest elevations (≥ 5.6%) increase risk [16].

Across large populations, pre‐gestational diabetes increases risk for all CHD subtypes, with the greatest excess for outflow‐tract defects the very spectrum observed here [17]. Moreover, recent studies, including reports in *JCI Insight*, suggest that certain genetic factors such as reduced function of the NOTCH1 gene may increase a fetus's vulnerability to the harmful effects of maternal hyperglycemia. These insights highlight the importance of achieving tight glucose control before and early in pregnancy to lower the risk of congenital heart defects in infants of mothers with diabetes [18].

Our case aligns published reports in several ways. For example, a study by Bernasconi et al. 2024, indicated that among 81 fetal dextrocardia cases (incidence 0.22%), 47% had situs solitus, 30% ambiguus, and 23% inversus. Cardiac malformations occurred in 66%, 96%, and 63% respectively; extracardiac defects in 31%, 21%, and 10%. There were 27 terminations, 11 deaths, and 43 survivors. They conclusively mentioned that most cases involve complex heart disease requiring detailed prenatal evaluation and counseling [19]. On the other hand, multiple contemporary studies link fetal CHD‐associated hydrops to markedly increased fetal/neonatal mortality, consistent with the intrauterine demise at 30 weeks here [11, 20]. In summary, this case illustrates a plausible diabetes‐related conotruncal malformation in the setting of isolated dextrocardia that progressed to cardiomegaly and hydrops with fetal demise. The literature consistently links pre‐gestational diabetes to outflow‐tract CHDs via hyperglycemia‐driven oxidative injury to neural crest–derived structures, and shows markedly worse outcomes once hydrops develops emphasizing the critical roles of preconception glycemic control and early targeted fetal cardiac screening to prevent, detect, and manage such high‐risk scenarios.

The principal limitation of this case is the absence of postmortem examination, which prevented definitive confirmation of the precise intra‐cardiac anatomy particularly regarding the suspected absent pulmonary valve variant of tetralogy of Fallot. Additionally, no genetic testing was performed, limiting assessment of possible syndromic or chromosomal contributions. As a single‐case observation, the findings cannot be generalized but highlight the importance of early screening, meticulous fetal surveillance, and maternal preconception glycemic control. Another limitation of this study is that 22q11.2 deletion and other genetic contributors remain unassessed [21, 22].

## Author Contributions

**Alireza Golbabaei:** conceptualization, investigation, methodology, supervision. **Vida Kahani:** data curation, supervision, validation. **Marzieh Mousivand:** data curation, software, visualization, writing – original draft, writing – review and editing.

## Funding

The authors have nothing to report.

## Ethics Statement

A written informed consent was obtained from the parents of the patient. The study was conducted according to the ethical declaration of Helsinki.

## Consent

A written informed consent was obtained from the parents of the patient.

## Conflicts of Interest

The authors declare no conflicts of interest.

## Data Availability

The data supporting the findings of this case report are available from the corresponding author upon reasonable request. Due to the sensitive nature of patient information and ethical considerations regarding privacy, the full dataset (including detailed echocardiographic images and clinical records) is not publicly available but can be shared in a de‐identified format after approval by the institutional ethics committee.
